# Optical coherence tomography in patients with Wilson's disease

**DOI:** 10.1002/brb3.3014

**Published:** 2023-04-16

**Authors:** Wei‐Qin Ning, Chun‐Xiao Lyu, Sheng‐Peng Diao, Ye‐Qing Huang, Ai‐Qun Liu, Qing‐Yun Yu, Zhong‐Xing Peng, Ming‐Fan Hong, Zhi‐Hua Zhou

**Affiliations:** ^1^ Department of Neurology Guangzhou Guangdong China

**Keywords:** duration, magnetic resonance imaging, optical coherence tomography, Wilson's disease

## Abstract

**Background:**

Morphological changes of retina in patients with Wilson's disease (WD) can be found by optical coherence tomography (OCT), and such changes had significant differences between neurological forms (NWD) and hepatic forms (HWD) of WD. The aim of this study was to evaluate the relationship between morphological parameters of retina and brain magnetic resonance imaging (MRI) lesions, course of disease, type of disease, and sexuality in WD.

**Methods:**

A total of 46 WD patients and 40 health controls (HC) were recruited in this study. A total of 42 WD patients were divided into different groups according to clinical manifestations, course of disease, sexuality, and brain MRI lesions. We employed the Global Assessment Scale to assess neurological severity of WD patients. All WD patients and HC underwent retinal OCT to assess the thickness of inner limiting membrane (ILM) layer to retinal pigment epithelium layer and inner retina layer (ILM to inner plexiform layer, ILM–IPL).

**Results:**

Compared to HWD, NWD had thinner superior parafovea zone (108.07 ± 6.89 vs. 114.40 ± 5.54 μm, *p* < .01), temporal parafovea zone (97.17 ± 6.65 vs. 103.60 ± 4.53 μm, *p* < .01), inferior parafovea zone (108.114 ± 7.65 vs. 114.93 ± 5.84 μm, *p* < .01), and nasal parafovea zone (105.53 ± 8.01 vs. 112.10 ± 5.44 μm, *p* < .01) in inner retina layer. Course of disease influenced the retina thickness. Male patients had thinner inner retina layer compared to female patients.

**Conclusion:**

Our results demonstrated that WD had thinner inner retina layer compared to HC, and NWD had thinner inner retina layer compared to HWD. We speculated the thickness of inner retina layer may be a potential useful biomarker for NWD.

## Graphical abstract

Our results demonstrated that WD had thinner parafovea zones and partial perifovea zones in inner retina layer compared to HC, NWD had thinner parafovea zones in inner retina layer compared to HWD. This study confirmed the thickness of inner retina layer may be a potential useful biomarker for NWD , OCT may be a useful tool for measuring the degree of neurodegeneration in WD patients and may play role in monitoring disease progression.

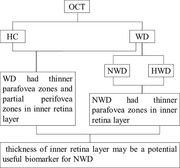

## 1 INTRODUCTION

Wilson's disease (WD) was first described by the British neurologist Kinnier Wilson in 1912. WD is an inherited autosomal recessive disorder that leads to copper accumulation in liver, brain, cornea, and other organs (Langwinska et al., 2017). The causative mutations affect the copper‐transporting P‐type ATPase ATP7B, which regulates hepatic copper metabolism, leading to impaired biliary excretion and toxic accumulation of copper (Albrecht, Müller, Ringelstein, et al., 2012; Albrecht, Müller, Südmeyer, et al., 2012). Clinical manifestations of WD include neurological, liver and psychosis symptoms, as well as Kayser–Fleischer ring (KFR) of the cornea (European Association for Study of Liver., 2012). KFR is typical for WD and represents the deposition of copper in posterior corneal layers. Sunflower cataracts (Rajappa et al., 2010), optic neuritis, optic disc pallor, and exotropia (Ala et al., 2007) are rare ophthalmological manifestations. Copper accumulates over the course of disease and causes different organs injury (Dusek, Litwin, et al., 2015; Dusek, Roos, et al., 2015; European Association for Study of Liver., 2012). Pathological injuries in brain are mainly located in basal ganglia, but copper may accumulate in other central nervous system (CNS) structures (Prashanth et al., 2005; Saatci et al., 1997).

Recent studies detected morphological changes of retina in WD patients by optical coherence tomography (OCT) (Albrecht, Müller, Ringelstein, et al., 2012; Albrecht, Müller, Südmeyer, et al., 2012; Langwinska et al., 2017, 2016; Svetel et al., 2021). Study found significant negative correlation between OCT parameters and UWDRS‐part II in WD (Langwinska et al., 2017); patients with abnormal brain magnetic resonance imaging (MRI) had thinner macular and retinal nerve fiber layer (RNFL) in WD (Langwinska et al., 2016). These indicated OCT parameters of retina may be a potential biomarker in WD. Recently, several biomarkers of CNS injury in WD were found. Studies indicated that serum neurofilament (sNFL) concentrations (Ziemssen et al., 2022), glial fibrillary acidic protein (Lin et al., 2021), microtubule‐associated protein tau (Lekomtseva et al., 2019), and ubiquitin C‐terminal hydrolase L1 (Shribman et al., 2021) may be a potential biomarker for CNS injury in WD. OCT is a fast, noninvasive, and dynamic technique and had been used in parkinsonian syndromes (Albrecht, Müller, Ringelstein, et al., 2012; Albrecht, Müller, Südmeyer, et al., 2012) and multiple sclerosis (Saidha et al., 2015). The newest OCT devices can depict retinal changes at nearly the cellular level (Saidha et al., 2011). On the basis of previous studies, we want to investigate the relationship between OCT parameters of retina and brain MRI lesions, course of disease, type of disease, and sexuality in Chinese WD patients and, finally, relate OCT parameters of retina to brain MRI lesions, course of disease, type of disease, and sexuality in WD patients and identify potential ophthalmological biomarkers of WD.

## 2 MATERIALS AND METHODS

All patients provided informed consent. We collected 46 WD patients admitted to the First Affiliated Hospital of Guangdong Pharmaceutical University from June 2021 to April 2022. Four patients were excluded because they could not cooperate with OCT examination because of severe tremors. A total of 42 WD patients were enrolled in this study, including 21 males and 21 females. All patients were Han nationality Chinese. All patients met the diagnostic criteria for WD (European Association for Study of Liver., 2012). Inclusion criteria: (1) defined WD and age ranged 15–60‐year old, (2) patients without hypertension and diabetes mellitus, (3) without ocular trauma on previous medical history, and (4) without other ophthalmic diseases. Exclusion criteria: (1) a contraindication to use 1% tropicamide, (2) patients who cannot undergo OCT examination due to severe involuntary movements (such as severe rest tremor), and (3) patients who were younger than 15‐year old. We also recruited 40 sexy‐age match healthy controls (HCs). Demographic and clinical characteristics of 42 WD patients are shown in Table 1.

**TABLE 1:** Clinical and demographic features of patients with neurological and hepatic form of Wilson's disease

Feature	Neurological form of disease	Hepatic form of disease	*p*‐Value
Number^a^	27 (64%)	15 (36%)	
Gender (male/female)^b^	16/11	4/11	.043^c^
Age (years)^b^	30.56 ± 10.12	30.53 ± 10.00	.995
Age at onset (years)^b^	19.96 ± 9.12	19.00 ± 11.02	.773
Age at diagnosis (years)^b^	20.39 ± 9.43	19.00 ± 11.08	.681
Disease duration (years)^b^	10.10 ± 6.79	11.40 ± 4.25	.513
Latency from diagnosis to treatments (years)^b^	1.01 ± 3.24	1.40 ± 4.33	.757
Treatment duration^b^	9.14 ± 7.05	11.13 ± 3.77	.323

^a^ Values presented as number of patients with percentage in brackets.

^b^ Values presented as means ± SDs in brackets.

^c^ *p <* .05.

**FIGURE 1:**
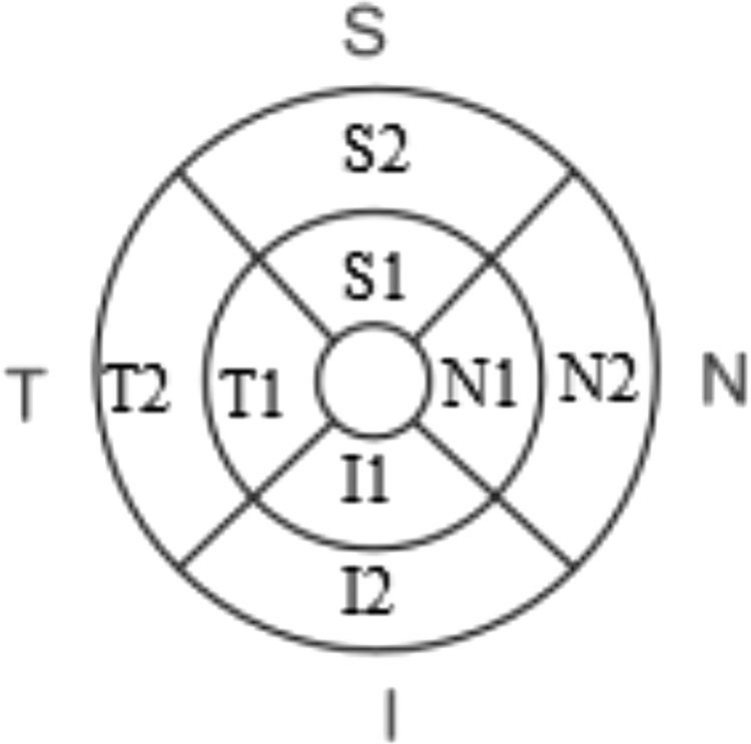
The areas of measurement are marked in an image of the fundus. The inner retina thickness and full retina thickness were measured in scans centered on the fovea. S1, T1, I1, N1 mean superior, temporal, inferior, and nasal parafovea zones in 3 × 3 mm^2^ centered on the fovea, S2, T2, I2, N2 mean superior, temporal, inferior, and nasal perifovea zone in 6 × 6 mm^2^ centered on the fovea.

According to clinical presentations, WD patients were divided into two groups, group of neurological (NWD) and group of hepatic (HWD). We employed the Global Assessment Scale to assess the neurological severity of WD patients (Aggarwal et al., 2009). Each participant underwent OCT using RTVue XR Avanti System (Optovue, Inc, Fremont, CA, USA) with AngioVue software (version 2018,1,1,63). All OCT scans were performed by the same experienced operator. The operator reviewed all OCT images, in order to identify segmentation errors, artifacts, and poor image quality, thus ensuring that only proper images were finally included in our study. The scanning areas used in this study were 3 × 3 mm^2^ (Angio Retina [3.0]) and 6 × 6 mm^2^ (HD Angio Retina [6.0]) both centered on the fovea (Figure 1).

After the completion of the scan process, the following measured parameters were recorded: Full retina thickness: inner limiting membrane (ILM) layer to retinal pigment epithelium layer, inner retina thickness: ILM to inner plexiform layer (ILM–IPL).

All patients underwent 3.0T brain MRI with a GE Discovery MR750, GE Medical Systems. All patients performed the following sequences: T1‐ and T2‐weighted scans, fluid‐attenuated inversion recovery and susceptibility‐weighted imaging. The presence of lesions on MRI in the following structures was collected: basal ganglia, cerebellum, brainstem, and other localizations in white matter.

### 2.1 Statistical analysis

To avoid internal correlations, data were analyzed by using mean values of measurements taken from both eyes. Quantitative variables were described using mean and standard deviation (M ± SD). Group differences of demographic categorical variables were tested using the Chi‐square test. Normality of data was visually inspected and tested with Shapiro–Wilk. Group comparisons of normally distributed variables were done with *T*‐test and non‐normally distributed data were assessed with Mann–Whitney *U*‐test. For comparison among groups, a one‐way analysis of variance with Bonferroni and Tukey HSD post hoc correction was performed for normally distributed data, to adjust for multiple comparisons. Pearson (*r*) and Spearman (ρor rho orr_s_) correlation coefficients were used to assess linear and nonlinear correlations, respectively, between any OCT parameters and neurological severity. Statistical analysis was performed using Statistical Package for the Social Sciences (SPSS version 23), and *p*‐values below .05 were considered statistically significant.

## 3 RESULT

A total of 42 WD patients (mean age 30.54 ± 9.83 years) were divided into 2 groups: NWD (11 females and 16 males, mean age 30.56 ± 10.12 years) and HWD (11 females and 4 males, mean age 30.53 ± 10.00 years). HC comprised 16 females and 24 males, with mean age of 30.42 ± 7.37 years.

In order to assess the relationship between OCT parameters and course of disease, all recruited patients were stratified into three groups: course of disease shorter than 5 years (<5‐year group), 5−10 years (5–10‐year group), and longer than 10 years (>10‐year group). There is no significant difference in the onset of age, age of diagnosis, course of disease, latency from diagnosis to treatment, and treatment duration between NWD and HWD.

### 3.1 OCT parameters in WD and HC

Compared to HC, WD had thinner superior parafovea zone, temporal parafovea zone, inferior parafovea zone, nasal parafovea zone, temporal perifovea zone, and nasal perifovea zone in inner retina layer (Table 2).

**TABLE 2:** Optical coherence tomography (OCT) parameters in Wilson's disease (WD), neurological form group (NWD), hepatic form group (HWD), and health controls (HC)

FRL (μm) M ± SD	HC (*n* = 40)	WD (*n* = 42)	HWD (*n* = 15)	NWD (*n* = 27)	*p* Value
S1	324.90 ± 13.85	326.36 ± 13.97	329.46 ± 16.21	324.64 ± 12.55	.503
T1	311.50 ± 10.61	311.15 ± 14.23	314.73 ± 15.85	309.16 ± 13.14	.388
I1	320.05 ± 11.94	321.33 ± 15.50	323.90 ± 18.44	319.90 ± 13.79	.618
N1	323.95 ± 11.75	324.60 ± 13.72	328.73 ± 13.74	322.31 ± 13.41	.289
S2	288.45 ± 16.88	287.36 ± 13.54	291.20 ± 14.41	285.24 ± 12.81	.457
T2	272.85 ± 17.97	269.75 ± 12.50	270.40 ± 14.14	269.38 ± 11.76	.652
I2	272.65 ± 16.16	276.00 ± 15.23	277.03 ± 14.69	275.42 ± 15.76	.601
N2	300.75 ± 18.47	297.94 ± 49.25	308.46 ± 16.60	292.09 ± 59.81	.378
IRL (μm) M ± SD					
s1	114.45 ± 6.32	110.33 ± 7.07^#^	114.40 ± 5.54	108.07 ± 6.89*^+^	**.000**
t1	103.15 ± 4.86	99.46 ± 6.69^#^	103.60 ± 4.53	97.16 ± 6.65*^+^	**.000**
i1	114.90 ± 4.47	110.57 ± 7.72^#^	114.93 ± 5.84	108.14 ± 7.65*^+^	**.000**
n1	112.50 ± 5.40	107.88 ± 7.80^#^	112.10 ± 5.44	105.53 ± 8.01*^+^	**.000**
s2	104.45 ± 10.01	99.78 ± 7.60^##^	103.33 ± 6.79	97.81 ± 7.42*	**.010**
t2	88.65 ± 7.31	85.53 ± 5.22^##^	86.90 ± 4.82	84.77 ± 5.37	.054
i2	100.95 ± 9.18	99.17 ± 6.86	101.73 ± 6.67	97.75 ± 6.67	.192
n2	122.45 ± 12.09	114.94 ± 19.93^##^	121.13 ± 7.26	111.50 ± 23.75*	**.026**

*Note*: Compared to HC, # means *p* < .01, ## means *p* < .05. Compared to HC, * means *p* < .01. Compared to HWD, + means *p* < .01. S1,T1,I1,N1 (s1,t1,i1,n1) mean superior, temporal, inferior, and nasal parafovea zones of full retina layer (inner retina layer) in 3 × 3 mm^2^ centered on the fovea, S2,T2,I2,N2 (s2,t2,i2,n2) mean superior, temporal, inferior, and nasal perifovea zones of full retina layer (inner retina layer) in 6 × 6 mm^2^ centered on the fovea. M ± S = means ± standard deviation. Statistically significant differences are represented in bold.

Abbreviations: FRL, full retina layer; IRL, inner retina layer.

### 3.2 OCT parameters in NWD, HWD, and HC

Compared to HC, NWD had thinner parafovea zones, superior perifovea zone, and nasal perifovea zone in inner retina layer (Table 2). Meanwhile, compared to HWD, NWD had thinner parafovea zones in inner retina layer (Table 2). There had no significant difference in OCT parameters between HWD and HC (Table 2).

### 3.3 OCT parameters in male and female

Previous studies showed that WD severity is associated with gender and the course of disease (Ferenci et al., 2019). In our study, we found that compared to female patients, male patients had thinner superior perifovea zone, inferior perifovea zone, and nasal perifovea zone in inner retina layer (Table 3).

**TABLE 3:** Optical coherence tomography (OCT) parameters in male and female

FRL (μm) M ± SD	Male (*n* = 20)	Female (*n* = 22)	Male vs. female *p*‐value
S1	328.76 ± 11.76	323.97 ± 15.79	.272
T1	314.59 ± 13.04	307.71 ± 14.85	.119
I1	324.57 ± 14.72	318.09 ± 15.94	.179
N1	327.54 ± 12.23	321.66 ± 14.77	.168
S2	285.45 ± 13.44	289.28 ± 13.68	.365
T2	271.59 ± 11.96	267.90 ± 13.04	.345
I2	275.90 ± 17.40	276.09 ± 13.13	.968
N2	303.34 ± 13.07	307.07 ± 15.75	.409
IRL (μm) M ± SD			
s1	110.09 ± 7.31	110.57 ± 7.00	.830
t1	99.61 ± 6.92	99.31 ± 6.62	.883
i1	110.02 ± 8.31	111.11 ± 7.25	.652
n1	108.16 ± 8.17	107.59 ± 7.60	.816
s2	97.16 ± 7.92	102.40 ± 6.44	**.024**
t2	85.26 ± 5.96	85.81 ± 85.81	.739
i2	96.64 ± 6.76	101.71 ± 6.11	**.015**
n2	114.32 ± 7.99	121.16 ± 7.05	**.005**

*Note*: S1,T1,I1,N1 (s1,t1,i1,n1) mean superior, temporal, inferior, and nasal parafovea in full retina layer (inner retina layer), S2,T2,I2,N2 (s2,t2,i2,n2) mean superior, temporal, inferior, and nasal perifovea in full retina layer (inner retina layer). M ± S = means ± standard deviation. Statistically significant differences are represented in bold.

Abbreviations: FRL, full retina layer; IRL, inner retina layer.

### 3.4 OCT parameters in different courses of disease

There is no significant difference between <5‐year group and 5–10‐year group. However, compared to <5‐year group, >10‐year group had thinner superior parafovea zone and nasal parafovea zone in full retina layer (Table 4). It seems that the thickness of full retina layer is associated with the course of disease, and patients with longer course of disease are more likely to occur morphological change of retina.

**TABLE 4:** Optical coherence tomography (OCT) parameters in different disease durations

FRL (μm) M ± SD	<5‐year group (*n* = 15)	5–10‐year group (*n* = 16)	>10‐year group (*n* = 11)	*p*‐Value
S1	335.28 ± 8.03	332.59 ± 12.51	322.25 ± 15.78^#^	.152
T1	318.64 ± 15.50	316.54 ± 10.98	307.39 ± 15.97	.262
I1	329.78 ± 14.89	326.13 ± 14.79	317.67 ± 17.13	.851
N1	334.00 ± 10.63	330.27 ± 12.14	320.78 ± 14.76^#^	.258
S2	297.71 ± 6.45	289.59 ± 15.13	285.96 ± 12.93	.059
T2	277.57 ± 13.62	272.00 ± 12.65	267.46 ± 12.75	.994
I2	282.28 ± 9.63	277.63 ± 12.85	275.17 ± 20.10	.411
N2	270.21 ± 119.35	311.72 ± 15.29	301.07 ± 14.62	**.002**
IRL (μm) M ± SD				
s1	114.07 ± 7.41	111.13 ± 6.28	110.39 ± 7.89	.556
t1	103.64 ± 6.79	98.81 ± 6.89	99.39 ± 7.38	.640
i1	115.71 ± 7.65	109.72 ± 7.96	110.21 ± 8.61	.854
n1	112.42 ± 7.92	109.18 ± 7.07	107.28 ± 8.40	.581
s2	103.35 ± 6.03	98.72 ± 9.27	100.71 ± 7.59	.480
t2	88.71 ± 5.77	84.45 ± 5.55	85.42 ± 5.63	.803
i2	101.07 ± 5.30	98.04 ± 5.61	98.57 ± 8.63	.642
n2	103.57 ± 46.08	118.95 ± 8.76	114.46 ± 22.44	**.007**

*Note*: Compared to <5‐year group, # means *p* < .05. S1,T1,I1,N1 (s1,t1,i1,n1) mean superior, temporal, inferior, and nasal parafovea zones of full retina layer (inner retina layer) in 3 × 3 mm^2^ centered on the fovea, S2,T2,I2,N2 (s2,t2,i2,n2) mean superior, temporal, inferior, and nasal perifovea zones of full retina layer (inner retina layer) in 6 × 6 mm^2^ centered on the fovea. M ± S = means ± standard deviation. Statistically significant differences are represented in bold.

Abbreviations: FRL, full retina layer; IRL, inner retina layer.

### 3.5 OCT parameters in different brain injury groups

Previous study had detected macular and RNFL were thinner, especially in patients with brain MRI lesions (Langwinska et al., 2016); we using a slightly different approach divided patients into three groups based on different brain MRI lesions: group 1—present of basal ganglia injury but absent of brainstem injury (ABI group), group 2—present of basal ganglia injury and brainstem injury (PBI group), and group 3—normal (no visual brain MRI lesions, MRI− group). In addition to brain stem and basal ganglia lesions, there are also other brain lesions in WD patients. A total of 13 patients had mild ventricular enlargement, and 10 patients had mild brain sulcus widening in our study. We only include the abnormal signals of basal ganglia and brainstem in our study.

In our study, there had no significant differences in OCT parameters between MRI− group and MRI+ group (ABI group + PBI group) (Table 5). PBI group had thinner nasal parafovea zone in inner retina layer compared to MRI− group (Table 6). However, there had no significant differences between ABI group and PBI group in OCT parameters (Table 6).

**TABLE 5:** Optical coherence tomography (OCT) parameters in MRI+ and MRI−

FRL (μm) M ± SD	MRI+ (*n* = 30)	MRI− (*n* = 12)	MRI+ vs. MRI− *p*‐value
S1	328.09 ± 15.12	327.00 ± 10.69	.869
T1	313.74 ± 14.86	309.41 ± 11.44	.510
I1	324.42 ± 16.06	318.00 ± 13.97	.373
N1	325.51 ± 14.66	327.16 ± 12.01	.800
S2	288.98 ± 14.16	287.41 ± 10.21	.801
T2	272.62 ± 13.34	264.83 ± 5.79	.175
I2	279.09 ± 17.18	269.25 ± 5.72	.180
N2	294.74 ± 60.79	306.16 ± 12.39	.377
IRL (μm) M ± SD			
s1	111.14 ± 6.70	114.58 ± 6.52	.281
t1	100.72 ± 6.13	99.50 ± 9.06	.689
i1	112.31 ± 6.67	110.33 ± 10.93	.564
n1	108.16 ± 7.84	113.16 ± 6.43	.157
s2	100.70 ± 6.80	103.58 ± 8.91	.381
t2	86.70 ± 4.84	85.50 ± 4.85	.599
i2	100.61 ± 6.52	98.75 ± 5.36	.521
n2	114.11 ± 23.84	121.16 ± 9.45	.486

*Note*: S1,T1,I1,N1 (s1,t1,i1,n1) mean superior, temporal, inferior, and nasal parafovea in full retina layer (inner retina layer), S2,T2,I2,N2 (s2,t2,i2,n2) mean superior, temporal, inferior, and nasal perifovea in full retina layer (inner retina layer). M ± S = means ± standard deviation.

Abbreviations: FRL, full retina layer; IRL, inner retina layer.

**TABLE 6:** Optical coherence tomography (OCT) parameters in different region injuries on visual brain magnetic resonance imaging (MRI)

FRL (μm) M ± SD	PBI group (*n* = 13)	ABI group (*n* = 17)	MRI− (*n* = 12)	*p*‐Value
S1	326.33 ± 9.31	328.59 ± 16.56	327.00 ± 10.69	.934
T1	310.161 ± 0.85	314.76 ± 15.90	309.41 ± 11.44	.640
I1	320.161 ± 0.47	325.64 ± 17.35	318.00 ± 13.97	.514
N1	321.33 ± 9.76	326.71 ± 15.78	327.16 ± 12.01	.700
S2	287.75 ± 13.05	289.33 ± 14.75	287.41 ± 10.21	.940
T2	273.52 ± 14.68	269.50 ± 6.99	264.83 ± 5.79	.319
I2	277.58 ± 13.29	279.52 ± 18.41	264.83 ± 5.79	.400
N2	305.161 ± 1.55	291.76 ± 68.77	306.16 ± 12.39	.795
IRL (μm) M ± SD				
s1	107.50 ± 3.39	112.19 ± 7.10	114.58 ± 6.52	.170
t1	97.41 ± 5.07	101.66 ± 6.18	99.50 ± 9.06	.361
i1	107.91 ± 4.04	113.57 ± 6.81	110.33 ± 10.93	.224
n1	103.00 ± 4.08	109.64 ± 8.09^#^	113.16 ± 6.43	.061
s2	99.00 ± 8.42	101.19 ± 6.42	103.58 ± 8.91	.555
t2	84.50 ± 3.33	87.33 ± 5.08	85.50 ± 4.85	.391
i2	100.75 ± 10.11	100.57 ± 5.45	98.75 ± 5.36	.815
n2	116.75 ± 8.29	113.35 ± 26.81	121.16 ± 9.45	.747

*Note*: Compared to MRI−, # means *p* < .05. S1,T1,I1,N1 (s1,t1,i1,n1) mean superior, temporal, inferior, and nasal parafovea zones of full retina layer (inner retina layer) in 3 × 3 mm^2^ centered on the fovea, S2,T2,I2,N2 (s2,t2,i2,n2) mean superior, temporal, inferior, and nasal perifovea zones of full retina layer (inner retina layer) in 6 × 6 mm^2^ centered on the fovea. ABI group = present of basal ganglia injury, but absent of brainstem injury, PBI group = present of basal ganglia injury and brainstem injury, MRI− = no visual brain MRI injury. M ± S = Means ± standard deviation.

Abbreviations: FRL, full retina layer; IRL, inner retina layer.

## 4 DISCUSSION

Our results indicated that WD had thinner parafovea zones and partial perifovea zones in inner retina layer compared to HC; NWD had thinner parafovea zones in inner retina layer compared to HWD. There was no significant difference in OCT parameters between HWD and HC. Our study found morphological change of retina occurs at NWD, and thinning of inner retina layer in HWD may be associated with neurological system injury. Therefore, the thickness of inner retina layer may be a potential biomarker for monitoring disease progression.

OCT is a noninvasive high‐resolution optical imaging technology; details of the principles of spectral‐domain OCT had described in previous study (Nassif et al., 2004). In the last 20 years, the development of OCT had fundamentally changed retinal diagnosis (Puliafito et al., 2010, 1995). Currently, due to a lack of direct access, human cerebral microvascular alterations are difficult to noninvasively monitor in vivo. Nevertheless, as the vasculature of retina and brain are similar in their anatomy and physiology (London et al., 2013), changes in the structure and function of brain and cerebral vessels may lead to changes in retina vessels and morphology. The embryological, structural, and functional continuity of retina with CNS makes the visual pathway a prime target for potential noninvasive investigations of neurodegeneration, such as neurodegeneration that occurs in course of Alzheimer's disease and multiple sclerosis (Greenberg et al., 2010).

Study showed that RNFL was thinner in WD compared to HC (Svetel et al., 2021). Our results indicated WD had thinner parafovea zones and partial perifovea zones in inner retina layer compared to HC. The reduction of RNFL thickness in WD reflects the degeneration of retinal ganglion cell axons and retinal ganglion cells themselves (Albrecht, Müller, Ringelstein, et al., 2012; Albrecht, Müller, Südmeyer, et al., 2012). It is likely to account for the observed reduced thickness of inner retina layer in our study. Neuronal degeneration as a consequence of axonal damage due to copper deposition along the optic nerve and tract is a plausible explanation for the abovementioned observations.

Langwinska et al. (2016) proved macular and RNFL were thinner, especially in patients with brain MRI lesions. In our study, we found there had no significant difference between MRI− group and MRI+ group (ABI group + PBI group). However, as we further divided the patients into three groups (ABI group, PBI group, and MRI− group) based on different brain MRI lesions, patients with more severe MRI lesions had thinner inner retina layer. We speculated thinning of inner retina layer is a slow process, aggravated with the aggravation of brain injury. Our results indicated that the thinning of inner retina layer in WD may be associated with neurologic injury.

Svetel et al. (2021) found the course of disease did not influence the RNFL thickness. However, in our study, we found that compared to <5‐year group, >10‐year group had thinner superior parafovea zone and thinner nasal parafovea zone in full retina layer. We speculated the thinning of full retina layer in patients with a longer course of disease, which may be associated with retinal neurodegeneration caused by excess copper. In summary, with a prolonging course of disease, the full retina layer gradually became thinner, which may indicate the full retina layer as a useful index for the tracking course of disease in WD.

A study of a large cohort of WD patients confirmed there was a gender effect in index patients: Hepatic presentation was more common in females, and neurologic presentation was more common in males (Ferenci et al., 2019). Agnieszka et al. conducted a higher sNFL concentration (a marker of neurodegeneration) was observed in men (estrogens are thought to have a neuroprotective effect in neurodegenerative diseases) (Antos et al., 2022). In our study, compared to females, males had thinner partial perifovea zones in inner retina layer. It conducted our study was consistent with previous studies, that male was more prone to neurological damage.

The retina consists of axons and glia without myelin and may be a good structure for visualizing the degree of neurodegeneration (MacLaren et al., 1996). Direct oxidative stress and apoptosis are principal pathways of copper toxicity in WD, resulting in cell death in the affected tissues (Nagasaka et al., 2006). To a certain extent, the excess copper was neutralized by antioxidants, but an elevated level of reactive oxygen species beyond a certain limit might induce undesirable oxidative damage in the cells (Steinebach et al., 1994; Gaetke et al., 200). Therefore, the observation of significant differences of inner retina layer in NWD and HWD supported previous study that chronic copper‐related degeneration affected unmyelinated fibers (Valenti, 2011).

## 5 CONCLUSION

Our results demonstrated that WD had thinner parafovea zones and partial perifovea zones in inner retina layer compared to HC, and NWD had thinner parafovea zones in inner retina layer compared to HWD. This study confirmed the thickness of inner retina layer may be a potential useful biomarker for NWD, and OCT may be a useful tool for measuring the degree of neurodegeneration in WD patients and may play role in monitoring disease progression.

## 6 LIMITATIONS

Our study had several limitations. First, we only compared OCT parameters in ABI group and PBI group, but WD also existed in other brain MRI lesions. We will apply brain semiquantitative scale performed by Dusek (Dusek, Litwin, et al., 2015; Dusek, Roos, et al., 2015) or volumetric studies assessing brain atrophy performed by Smolinski et al. (2019) in future. Second, because of the small sample size of this study, we cannot conclude the specific significance of thinning of inner retina layer and full retina layer in WD. We will enlarge the sample and follow up to compare the changes of OCT parameters before and after treatment.

## AUTHOR CONTRIBUTIONS

*Data collection; analysis and manuscript writing*: Wei‐Qin Ning. *Data collection and manuscript writing*: Chun‐Xiao Lyu. *Study design; manuscript revision and interpretation of data*: Zhi‐Hua Zhou and Ming‐Fan Hong. *Data collection; analysis and interpretation of data*: Sheng‐Peng Diao, Ye‐Qing Huang, Ai‐Qun Liu, Qing‐Yun Yu, and Zhong‐Xing Peng. All authors read and approved the final manuscript.

## CONFLICT OF INTEREST STATEMENT

The authors declare that they have no conflict of interest.

### PEER REVIEW

The peer review history for this article is available at https://publons.com/publon/10.1002/brb3.3014.

## Data Availability

The authors confirm that all data underlying the findings are fully available without restriction. All relevant data are within the paper.
